# Host Defense Versus Immunosuppression: Unisexual Infection With Male or Female *Schistosoma mansoni* Differentially Impacts the Immune Response Against Invading Cercariae

**DOI:** 10.3389/fimmu.2018.00861

**Published:** 2018-04-24

**Authors:** Martina Sombetzki, Nicole Koslowski, Anne Rabes, Sonja Seneberg, Franziska Winkelmann, Carlos Fritzsche, Micha Loebermann, Emil C. Reisinger

**Affiliations:** Division of Tropical Medicine and Infectious Diseases, Center of Internal Medicine II, University Medical Center Rostock, Rostock, Germany

**Keywords:** *Schistosoma mansoni* infection, air pouch, inflammation, immune suppression, single-sex infection, granulomatous inflammation

## Abstract

Infection with the intravascular diecious trematode *Schistosoma spp*. remains a serious tropical disease and public health problem in the developing world, affecting over 258 million people worldwide. During chronic *Schistosoma mansoni* infection, complex immune responses to tissue-entrapped parasite eggs provoke granulomatous inflammation which leads to serious damage of the liver and intestine. The suppression of protective host immune mechanisms by helminths promotes parasite survival and benefits the host by reducing tissue damage. However, immune-suppressive cytokines may reduce vaccine-induced immune responses. By combining a single-sex infection system with a murine air pouch model, we were able to demonstrate that male and female schistosomes play opposing roles in modulating the host’s immune response. Female schistosomes suppress early innate immune responses to invading cercariae in the skin and upregulate anergy-associated genes. In contrast, male schistosomes trigger strong innate immune reactions which lead to a reduction in worm and egg burden in the liver. Our data suggest that the female worm is a neglected player in the dampening of the host’s immune defense system and is therefore a promising target for new immune modulatory therapies.

## Introduction

Infection with the intravascular trematode *Schistosoma spp*. remains a serious tropical disease and public health problem in the developing world. It is responsible for up to 300,000 deaths per year and 36 million disability-adjusted life years (1–3). *Schistosoma mansoni*, which causes intestinal schistosomiasis, accounts for around 80 million infections in Africa, the Near East and South America (4).

During infection, the host’s immune system is faced with different developmental stages of the parasite, from skin-penetrating cercarial larvae, juveniles which pass through the heart and lungs, and finally egg-producing adult worm pairs which inhabit the mesenteric vasculature. The adults produce up to 300 tissue-damaging eggs per day (5). Most of the eggs are flushed into the liver where they become entrapped within the hepatic sinusoids and provoke a sustained inflammatory-driven process. Some eggs penetrate the blood vessel walls, inducing granulomatous inflammation in the connective tissues. A small proportion finally migrates into the gut lumen to be released *via* feces. After some years, the accumulating eggs cause pronounced intestinal and hepatic fibrosis, which in turn leads to portal hypertension and its sequels—including variceal bleeding and ascites (6, 7).

The innate immune responses which are the first line of defense against invading *S. mansoni* cercariae are mediated by the skin. The skin constitutes the immune-regulatory checkpoint that guides immune responses and therefore determines parasite survival and the extent of parasite morbidity. First exposures to cercariae are associated with the development of Th1 responses in the skin. The rapid recruitment of neutrophils and eosinophils and the activation of complement factors have been shown to efficiently kill invading *Schistosoma* larvae (8, 9).

The parasites then evolve a range of mechanisms to actively dampen the host’s immune response and promote their own survival. Repeated infections in mice have been shown to trigger changes in inflammatory cell composition in skin-draining lymph nodes, CD4^+^ T cell hypo-responsiveness, changes in the local cytokine environment, and alternative activation of macrophages and dendritic cells (10, 11). Mice exposed multiple times to cercariae display a shift toward Th2-type activity, which primes the immune responses to adult worms, parasite eggs, and granuloma formation (12, 13).

Several studies have demonstrated that the sex of schistosomes affects the host’s immune response and disease progression (14–16), but whether it modulates the early immune response in the skin as well has remained elusive to date. We combined a murine single-sex infection system with an air pouch model to investigate immune reactions to injected cercariae. We were able to show that male and female schistosomes have opposing immune modulatory roles during early reinfection and differentially impact the host’s defense mechanisms.

## Materials and Methods

### Ethics Statement

Animal experiments were performed in strict accordance with the regulations of the German Society for Laboratory Animal Science and with the European health guidelines issued by the Federation of Laboratory Animal Science Associations. The protocol was approved by the local committee on animal care and use (7221.3-1.1-008/13, 7221.3-1-047/16, 7221.3-2-024/15-2). All efforts were made to minimize animal suffering.

### *S. mansoni* Infection Model

*Schistosoma mansoni* (Belo Horizonte strain) was kept in a life cycle using *Biomphalaria glabrata* fresh water snails (Brazilian strain) as intermediate hosts and 6- to 8-week-old female NMRI mice as definitive hosts, as previously described (17).

To obtain either male or female cercariae for subsequent infection of mice, *B. glabrata* snails were exposed to single *S. mansoni* miracidia, and cercariae were harvested 6 weeks later (18). The sex of the cercariae was determined by DNA amplification of sex-related chromosome segments using female-specific primers as previously described (14, 18–21). The study designs were illustrated in a graphic overview (Figure S1 in Supplementary Material). To distinguish the study design “air pouch” from “bisexual reinfection” different designations were used. For the study “air pouch,” we used lower case for the reinfection step (…/mf). For the study “bisexual reinfection,” we used upper case (…/MF). The following study designs were used.

“Air pouch” study design: 6-week-old female C57BL/6 mice were percutaneously infected with 100 *S. mansoni* cercariae, either male only (m), female only (f), or both sexes (mf). A further group was left uninfected (–). On day 37 postinfection (p.i.), air pouches were created by subcutaneously injecting 4 ml sterile air into a shaved skin site on the back of each mouse under isoflurane anesthesia. Air pouches were reinflated with 2 ml sterile air after 2 and 4 days (22). On day 42 p.i., 1 ml PBS or 50 *S. mansoni* cercariae of both sexes (in 1 ml PBS) were injected into the air pouch. Groups were designated as –/PBS (*n* = 5), –/mf (*n* = 10), m/mf (*n* = 10), f/mf (*n* = 10), and mf/mf (*n* = 10). The symbol ahead of the slash indicates the nature of the first infection step. The symbol after the slash indicates the injection of mixed sex cercariae into the air pouch. Mice were sacrificed 6 hours later *via* cervical dislocation under isoflurane anesthesia. The pouches were rinsed with 3 ml PBS, kneaded, and exudates were collected. After centrifugation, exudate cells were counted in a Neubauer counting chamber and analyzed using flow cytometry. Cell-free supernatants were stored at −80°C for further analysis.

“Antibodies and gene expression” study design: 6-week-old female C57BL/6 mice were percutaneously infected with 100 *S. mansoni* cercariae, either male only (m, *n* = 11), female only (f, *n* = 11), or both sexes (mf, *n* = 10), or left untreated (–, *n* = 10). Mice were sacrificed 6 weeks p.i. *via* cervical dislocation under isoflurane anesthesia. Spleens and blood serum were collected for further analysis.

“Bisexual reinfection” study design: 6-week-old female C57BL/6 mice were percutaneously infected with 100 *S. mansoni* cercariae, either male only (m), female only (f), or both sexes (mf). A further group was left uninfected (–). Six weeks later, mice were reinfected with 50 or 150 *S. mansoni* cercariae of both sexes to assess inflammatory immune response and disease progression and to determine worm and egg burden, respectively. Groups were designated as – (*n* = 5), –/MF (*n* = 8), m/MF (*n* = 8), f/MF (*n* = 8), and mf/MF (*n* = 8). The symbol ahead of the slash indicates the nature of the first infection step. The symbol after the slash indicates the second bisexual infection step. Mice were sacrificed *via* cervical dislocation under ketamine/xylazine anesthesia 14 weeks postinfection.

### Flow Cytometry

Single-cell suspensions from air pouch exudates were stained for surface markers in ice-cold PBS (0.5% BSA and 0.1% sodium azide) for 30 min and fixed with 4% paraformaldehyde for 10 min at room temperature. The following fluorochrome-tagged antibodies were used: anti-CD45-PerCP, anti-CD11b-PE, anti-CD11c-Alexa488, anti-Gr-1-PE-Cy7, anti-F4/80-APC, anti-B220-PE-Cy7, anti-CD3-APC, anti-CD4-FITC, and anti-CD8-APC-Cy7 (all purchased from BioLegend, USA) and anti-CD44-PE, anti-62L-APC-Cy7, anti-B220-APC-Cy7, anti-PD-L2-APC, anti-CD73-PE, anti-CD80-PerCP-Cy5.5-APC (all purchased from BD Bioscience). CD45^+^ cells were differentiated by gating on the following cell populations: dendritic cells (CD11b^+^/CD11c^+^), macrophages (CD11b^+^F4/80^+^), neutrophils (CD11b^+^/Gr-1^+^F4/80^−^), eosinophils (CD11b^+^/Gr-1^+^F4/80^+^, FSC^low^), Gr1^+^ inflammatory monocytes (CD11b^+^/Gr-1^+^F4/80^+^, FSC^high^), T cells (CD3^+^/CD4^+^ or CD3^+^/CD8^+^), B cells (B220^+^), memory T cells (CD3^+^/CD44^high^/CD62L^low^), and memory B cells (B220^+^/PD-L2^+^/CD73^+^/CD80^+^). Dead cells were stained using Zombie Red™ Fixable Viability Kit (BioLegend, USA) according to the manufacturer’s instructions. Data were obtained using a FACS Verse flow cytometer (BD Bioscience) and analyzed using FlowJo software (v10.0.7, Tree Star Inc., CA, USA) (for a list of all antibodies and enzyme-linked immunosorbent assays used in this study, see Table S1 in Supplementary Material).

### Quantification of Cytokines and Chemokines

Concentrations of cytokines (IFN-γ, TNF-α, IL-1β, IL-12p70, IL-4, IL-5, IL-13, IL-10) and chemokines (RANTES, Eotaxin, MIP-1α, MIP-1β, MCP-1, CXCL-1, CXCL-2, CXCL-10) in the air pouch exudate were determined using ProcartaPlex™ Luminex Multiplex Immunoassay (eBioscience, Germany) according to the manufacturer’s instructions. TGF-β serum concentrations were analyzed using a commercially available ELISA kit (TGF beta-1 Human/Mouse Uncoated ELISA Kit, Life Technologies, Germany) according to the manufacturer’s instructions.

### Quantification of Schistosoma-Specific Immunoglobulins

Six weeks after infection, immunoglobulin E (IgE) and immunoglobulin G (IgG1, IgG2b) subclasses specific for to soluble *S. mansoni* adult worm antigens were quantified in serum using an in-house ELISA as previously described (14).

### Quantitative Real-time PCR (RT-PCR) Analysis

Six weeks after infection, total RNA was isolated from snap-frozen spleens (RNeasy Plus Mini Kit, Qiagen, Germany) and reversely transcribed into cDNA using High-Capacity cDNA Reverse Transcriptase Kit (ThermoFisher, Germany) according to the manufacturer’s instructions. RT-PCR was performed using the following TaqMan Gene Expression Assays: Foxp3 Mm00475162; Il10 Mm01288386, Ctla4 Mm00486849; Rnf138 Mm01158179; Il33 Mm00505403 and Il1rl1 Mm00516117 (ThermoFisher, Germany). Cycling was performed on the 7900HT Fast RT-PCR System under the following reaction conditions: 50°C for 2 min followed by 95°C for 10 min, 45 cycles at 95°C for 15 s, and at 60°C for 1 min. Gene expression values were normalized to the endogenous reference gene GAPDH (Rodent GAPDH control reagent, ThermoFisher, Germany) and presented as normalized, expression values relative to naive controls.

### Assessment of Parasite Burden

Total egg numbers were assessed by microscopic evaluation (100-fold magnification) of weighted liver fractions (crushing preparation). Parasites were recovered from the portal system using the liver perfusion technique previously described (17), and worm pairs subsequently counted.

### Pathology

Fourteen weeks after infection, harvested livers and spleens were weighed and the ratio of liver and spleen weight, respectively, to body weight was determined. Serum biochemistry for alanine aminotransferase (ALT), aspartate aminotransferase (AST), and alkaline phosphatase (AP) was performed using UniCel^®^ DxC 800 Synchron^®^ Clinical System (Beckman Coulter GmbH).

For histological evaluation, half of the right liver lobe was fixed in 10% neutral buffered formalin solution (Sigma Aldrich, Germany) and embedded in paraffin. Thin sections of 5 µm were stained with either hematoxylin/eosin (H&E) or sirius red (SR). Granuloma size was determined using ImageJ software (v1.47v; National Institutes of Health, USA). The extent of hepatic fibrosis was analyzed in thin sections stained for collagen (SR). The SR-positive areas were assessed using ImageJ software (v1.47v; National Institutes of Health, USA).

### Statistics

Statistical analysis was performed using GraphPad Prism 5.0 (GraphPad Software, La Jolla, CA, USA). Values are expressed as mean + SEM. Normal distribution was tested using the D’Agostino and Pearson Omnibus Normality Test. Normally distributed samples were compared using ANOVA followed by a Bonferroni *post hoc* test. Non-normally distributed samples were compared using the Kruskal–Wallis test followed by a Dunn’s *post hoc* test. For all statistical analyses, *p* values <0.05 were considered significant. *p < 0.05, **p < 0.01, ***p < 0.001, n.s., not significant.

## Results

### Female Schistosomes Suppress the Recruitment of Innate Immune Cells

**Figure 1:**
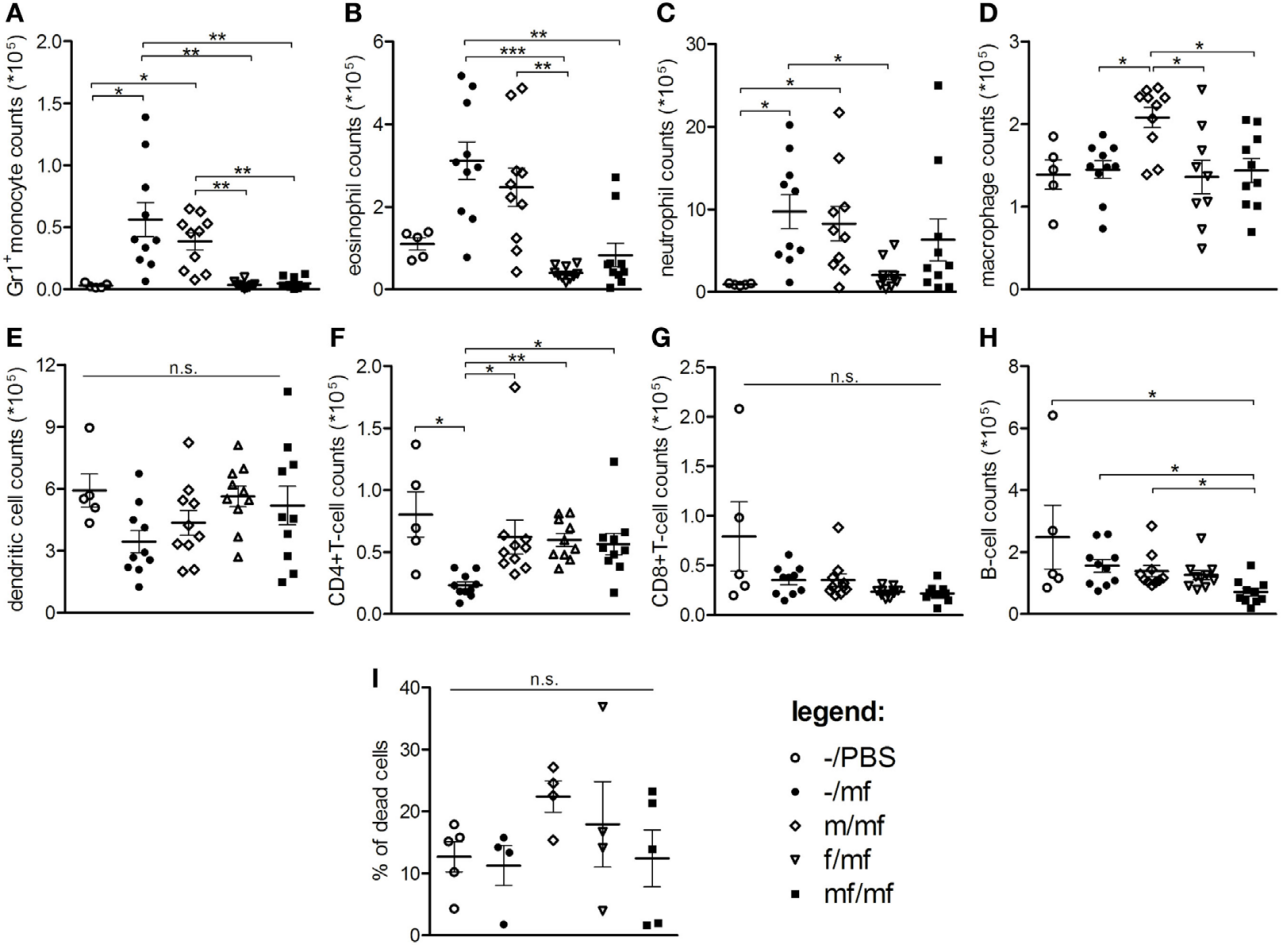
Flow cytometry: Female schistosomes suppress the recruitment of innate immune cells. Total numbers of **(A)** Gr1+ inflammatory monocytes, **(B)** eosinophils, **(C)** neutrophils, **(D)** macrophages/resident monocytes, **(E)** dendritic cells, **(F)** CD4+-T cells, **(G)** CD8+-T cells, **(H)** B cells, and **(I)** percentage of dead cells were analyzed in air pouch exudates. Data from individual experiments are depicted as individual symbols, lines represent mean ± SEM; *n* = 5 or 10 mice each group; *p < 0.05, **p < 0.01, ***p < 0.001, n.s., not significant.

To investigate whether the sex of adult *S. mansoni* living in the intestine impacts the early dermal immune defense reaction to invading cercariae, cercariae were injected into air pouches in mice carrying adult female worms, adult male worms, or worm pairs. Cell types and counts in the air pouches were determined by flow cytometry. Cell counts and percentages of innate effector cells such as Gr1^+^ monocytes, eosinophils, and neutrophils were highest in groups –/mf and m/mf. In comparison, in groups f/mf and mf/mf, cell counts of innate effector cells were low and comparable to the healthy control –/PBS (Figures 1A–C; Figures S2B,D in Supplementary Material). Macrophage numbers and percentages were higher in the m/mf group than the other groups (Figure 1D; Figure S2E in Supplementary Material). Comparable numbers of dendritic cells were found in the exudates of all mice (Figure 1E). Numbers of CD4^+^ T cells were significantly higher in all preinfected mice (m/mf, f/mf, mf/mf) than in group –/mf mice (Figure 1F), while the number of B cells was lowest in group mf/mf (Figure 1H). However, the sex of schistosomes had no impact on total numbers of CD4^+^ and CD8^+^ T cells, B cells or memory T and B cells in the air pouch (Figures 1F–H; Figure S3 in Supplementary Material). Neither percentages of dead cells nor total cell numbers in air pouch exudates were significantly different between groups (Figure 1I; Figure S2A in Supplementary Material). These data demonstrate that female adult worms, and to a lesser extent worm pairs, efficiently suppress the early recruitment of innate immune cells in response to injected cercariae.

### Female Schistosomes Reduce Chemokine and Cytokine Production

**Figure 2:**
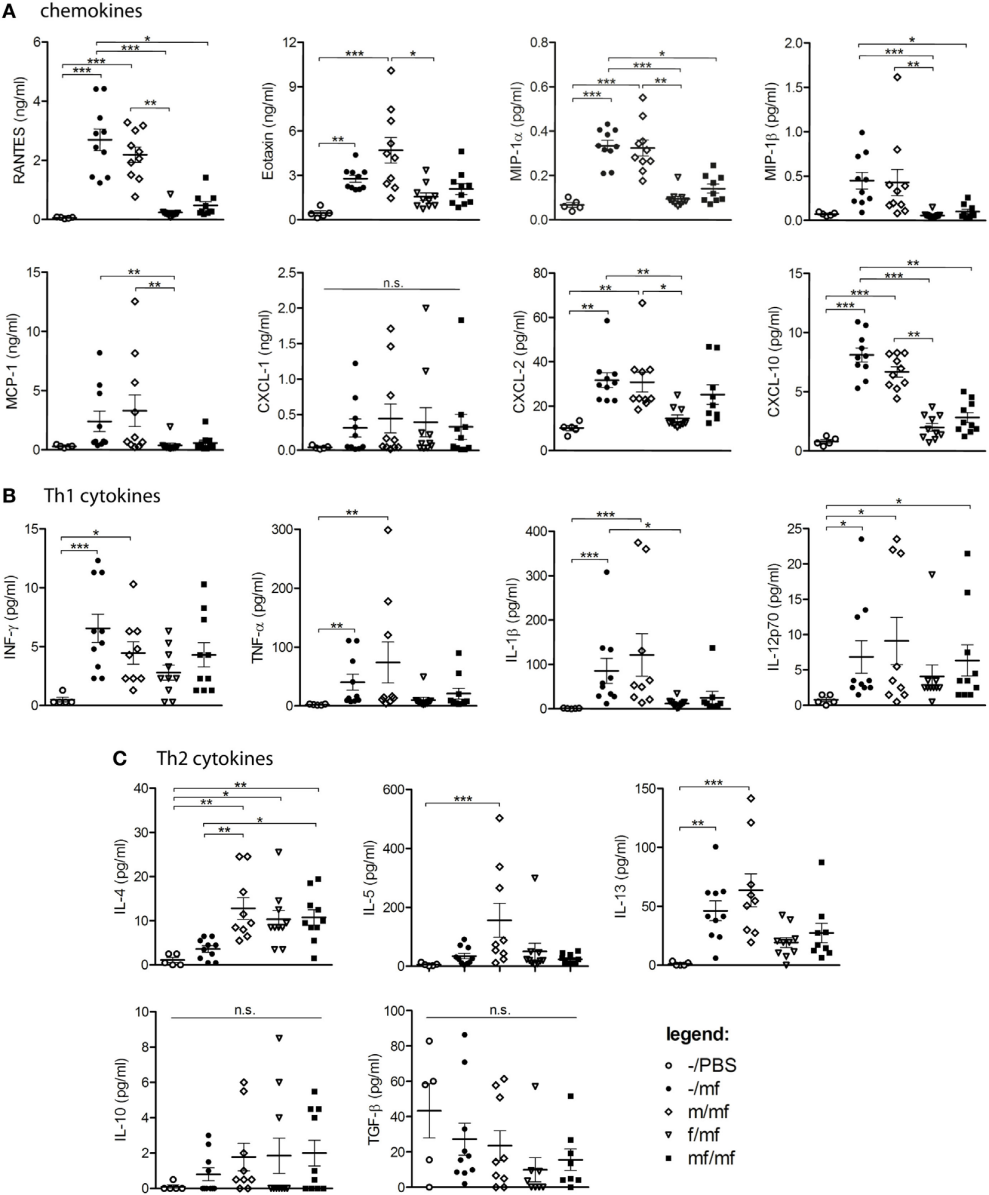
Multiplex analysis: female schistosomes reduce chemokine and cytokine production. Concentrations of **(A)** chemokines (RANTES, Eotaxin, MIP-1α, MCP-1, CXCL-1, CXCL-2, CXCL-10), **(B)** Th-1 cytokines (INF-y, TNF-α, IL-1β, IL-12p70), and **(C)** Th2-cytokines (IL-4, IL-5, IL-13, IL-10, TGF-β) were analyzed in air pouch exudates of *Schistosoma mansoni*-infected mice. Data from individual experiments are depicted as individual symbols, lines represent mean ± SEM; *n* = 5 or 10 mice each group; *p < 0.05, **p < 0.01, ***p < 0.001, n.s., not significant.

Chemokines and cytokines were determined in the air pouch exudates in order to provide insights into inflammatory mediators. Levels of RANTES, Eotaxin, MIP-1α, MIP-1β, MCP-1, and CXCL10 were significantly increased in groups –/mf and m/mf compared to the uninfected control –/PBS. In contrast, chemokine levels in groups f/mf and mf/mf were comparable to those of the uninfected control group –/PBS (Figure 2A).

Levels of the Th1 cytokines IFN-γ, TNF-α, IL-1β, and IL-12p70 were significantly increased in groups –/mf and m/mf compared to the uninfected control –/PBS and were lowest in groups f/mf and mf/mf, which exhibited comparable levels to –/PBS in the case of TNF-α and IL-1β (Figure 2B). The Th2 cytokine IL-4 was significantly elevated in all preinfected mice (groups m/mf, f/mf, mf/mf), whereas IL-5 was only elevated in group m/mf. IL-13 was significantly elevated in groups –/mf and m/mf in comparison to the other infected groups, while the regulatory cytokines IL-10 and TGF-β were not elevated in any of the groups (Figure 2C). These observations suggest that preinfection with female schistosomes and preinfection with worm pairs impedes the production of chemokines in response to a subsequent cercariae injection, resulting in a decline in the chemotactic attraction of immune cells.

### Male Schistosomes Increase the Production of Protective IgG1 and IgG2b Antibodies

**Figure 3:**
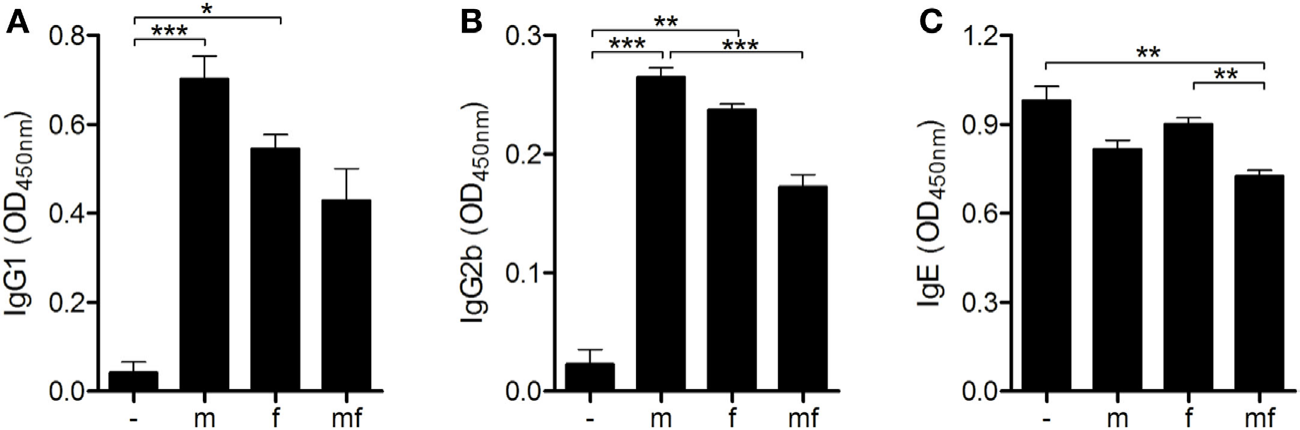
Male schistosomes increase the production of protective IgG1 and IgG2b antibodies. Six weeks p.i., **(A)** IgG1, **(B)** IgG2b, and **(C)** immunoglobulin E (IgE) serum levels of *Schistosoma mansoni*-infected mice were quantified by ELISA. Data are represented as mean + SEM; *n* = 5 or 10 mice each group; *p < 0.05, **p < 0.01, ***p < 0.001.

The next objective was to examine the role of *S. mansoni* sex on humoral adaptive immune responses. Levels of *S. mansoni*-specific IgG1, IgG2b, and IgE were measured in the blood of all mice before air pouches were prepared. IgG1 and IgG2b were found in all infected mice, with the highest levels detected in group m. However, levels of specific IgE were not elevated compared with uninfected control mice (Figure 3).

### Female Schistosomes Upregulate Anergy-Related Gene Expression

**Figure 4:**
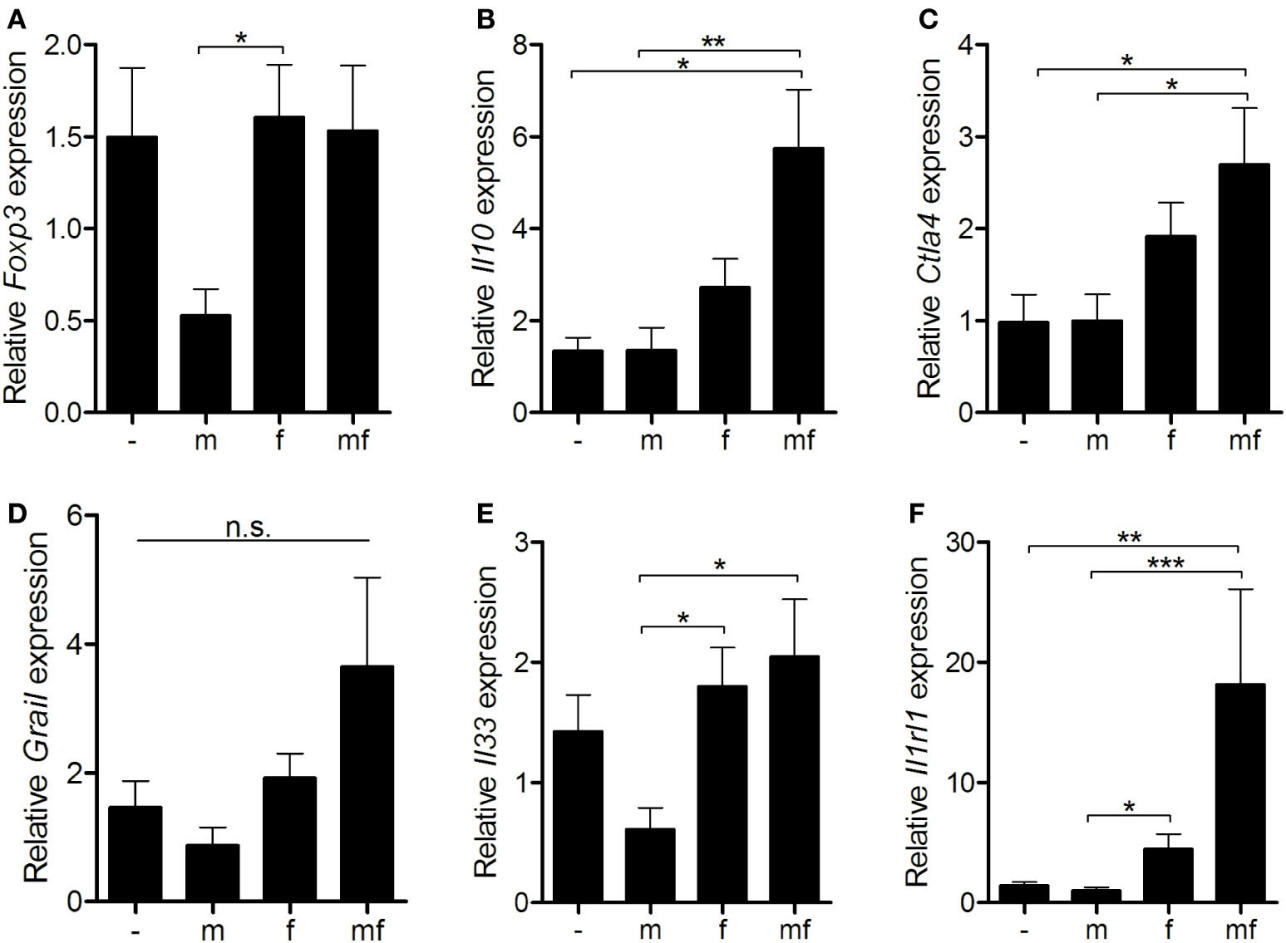
Female schistosomes upregulate anergy-related gene expression. Six weeks p.i. relative expression of **(A)** *Foxp3*, **(B)** *Il10*, **(C)** *Ctla4*, **(D)** *Grail*, **(E)** *Il33*, and **(F)** *Il1rl1* in spleens was determined by real-time PCR. Data are presented as mean + SEM; *n* = 10–11 mice each group; *p < 0.05, **p < 0.01, ***p < 0.001, n.s., not significant.

Since mice carrying female schistosomes showed a diminished dermal immune response to invading cercariae, expression levels of regulatory immunosuppressive genes were analyzed in uninfected (–), male only (m) or female only (f) or bisexually (mf) infected mice. *Fork head protein 3* (*Foxp3*), a marker for regulatory T cells, was significantly downregulated following infection with male only cercariae (group m) in comparison to the others (Figure 4A). In group f, the expression levels of *Il10* and *cytotoxic T-lymphocyte-associated protein 4* (*Ctla4*) are slightly increased compared to group m and uninfected control mice (Figures 4B,C). The highest expression levels of *Il10, Ctla4*, and *gene related to anergy in lymphocytes* (*Grail*) were found in bisexually infected mice (group mf), possibly as a result of parasite eggs already present in these mice (Figure 4D). Moreover, mRNA expression of immune modulatory *Il33* and its receptor *Il1rl1*, known to be expressed on activated Treg cells, were significantly elevated in group f and mf mice compared to group m (Figures 4E,F). Overall, the data indicate that female worms upregulate expression levels of regulatory *Il10* and *Ctla4* to a greater extent than male worms, thus promoting the establishment of an immunosuppressive environment.

### Primary Infection With Male Schistosomes Reduces Worm and Egg Burden but Has No Impact on Liver Fibrosis

A second experiment was performed to investigate whether immune responses to male and female schistosomes differentially impact parasite burden and disease progression. Mice were initially infected with male, female or mixed *S. mansoni* cercariae. Six weeks later bisexual reinfection was performed in a water bath containing a mixture of male and female *S. mansoni* cercariae. Eight weeks later, parasite loads and hepatic fibrosis levels were analyzed (Figure S1 in Supplementary Material).

**Figure 5:**
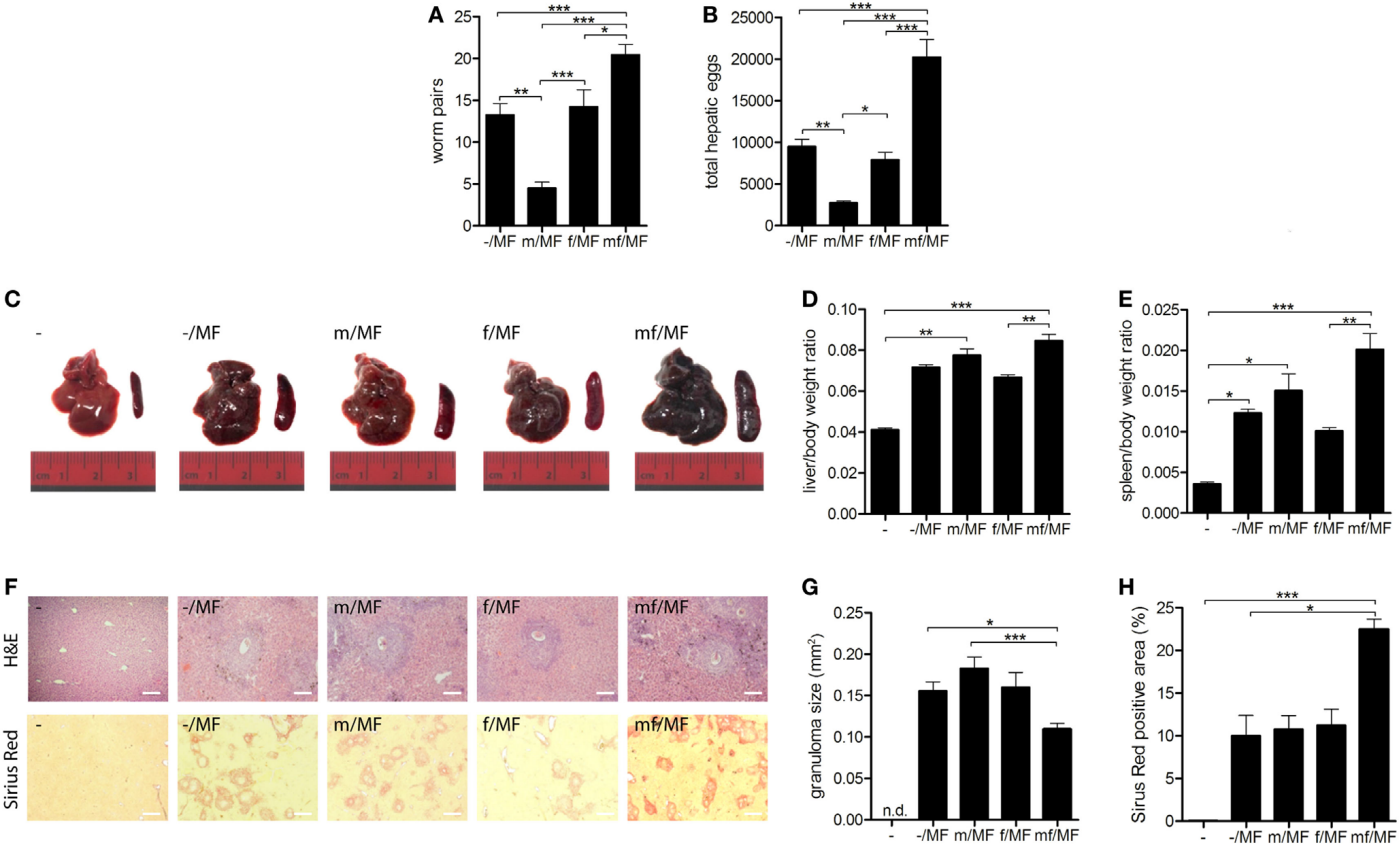
Primary infection with male schistosomes reduces worm and egg burden but has no impact on liver fibrosis. Six weeks p.i. mice were reinfected with **(A,B)** 150 or **(C–H)** 50 *Schistosoma mansoni* cercariae of both sexes (MF) and analyzed 8 weeks later. **(A)** Worm and **(B)** hepatic egg burden were quantified. **(C)** Representative images of livers and spleens. Relative organ size of **(D)** livers and **(E)** spleens expressed as a ratio to body weight. **(F)** Representative images of liver sections stained with hematoxylin/eosin and sirius red (magnification 100-fold). **(G)** Size of hepatic granulomas and **(H)** sirius red-stained area were quantified in Image J. Data are represented as mean + SEM; *n* = 5–8 mice each group; scale bar represents 200 µm. *p < 0.05, **p < 0.01, ***p < 0.001.

The numbers of worm pairs and eggs were found to be significantly reduced in group m/MF compared to the other groups. The highest numbers of worm pairs and hepatic eggs were seen in group mf/MF (Figures 5A,B). Macroscopic evaluation of all infected mice revealed enlarged livers and spleens with rough surfaces and visible nodules. These organ alterations were most prominent in mice of group mf/MF (Figure 5C). Body weight-adjusted liver and spleen sizes of mice preinfected with female cercariae (f/MF) were lower than those of the other infected groups (–/MF, m/MF, mf/MF) (Figures 5D,E). Serum levels of the liver enzymes AST and ALT were slightly elevated in groups –/MF, f/MF, and m/MF compared to the others (Figure S4 in Supplementary Material), whereas AP levels were decreased in all infected groups when comparing to uninfected mice.

Granuloma formation and fibrosis, shown by H&E and SR staining, were detected in all infected groups (Figure 5F). Hepatic granulomas did not differ in size between groups –/MF, m/MF, and f/MF, but were significantly smaller in group mf/MF (Figure 5G). Levels of hepatic fibrosis were the same in groups –/MF, m/MF, and f/MF and significantly higher in group mf/MF (Figure 5H).

These results suggest that a strong innate immune response in the skin of mice initially infected with male worms leads to a significant reduction in worm pairs and egg burden after reinfection. However, the reduction of parasite load in group m/MF had no effect on granuloma size or hepatic fibrosis.

## Discussion

In this study, we demonstrate that male and female schistosomes play different roles in modulating the host’s immune response. Female schistosomes suppress early innate immune responses to invading cercariae in the skin and upregulate anergy-associated genes, whereas male schistosomes trigger strong innate immune reactions. We further show that the strong immune responses within the skin provoked by male schistosomes result in the reduction of worm and egg burden in the liver.

Previous studies have shown an increase in liver and spleen weights and higher numbers of blood leukocytes in mice infected with male schistosomes, suggesting that male worms are more immunogenic. However, the underlying mechanisms and the effect of the sex of schistosomes on subsequent infections were not investigated (15). In another study, no differences between the sizes of lung granulomas were found in mice that were preinfected with male, female, or both sexes of *S. mansoni* cercariae and subsequently challenged with intravenously injected *Schistosoma* eggs (12). We recently provided evidence of the immune-suppressive potential of female worms by noting smaller perioval granuloma sizes, lower levels of inflammation, and less hepatic fibrosis in mice initially infected with female schistosomes prior to a secondary bisexual infection (14).

An appropriate innate immune response in the skin plays an important role in determining immune responses to developing *Schistosoma* larvae (13) and is required to combat such larvae (8, 23). Using an inflammatory air pouch model, we have shown that in contrast to male worms, females and worm pairs suppress the inflammatory immune response within the skin. This is reflected by lower levels of chemokines and cytokines in air pouch exudates, which in turn results in declined chemotactic attraction of immune cells. Cell numbers can be affected not only by local cell proliferation upon stimulation, but also by cell death. The air pouch model used in our study provides a localized environment in which to study and quantify cell recruitment and the production of chemokines and cytokines following injection of an inflammatory stimulus, but it is not appropriate for studying cell proliferation in the surrounding tissues. An increase of innate immune cells is mediated by active recruitment of these cells to the site of infection, a process initiated by the production of chemokines. As described elsewhere, *S. mansoni* infection elicits an initial influx of neutrophils into the skin, followed by eosinophils, macrophages, and dendritic cells (8, 24). The latter can be derived from infiltrating monocytes (25). As the percentage of dead cells did not vary between our experimental groups, we suggest that the reduction in the recruitment of innate immune cells in groups f/mf and mf/mf is mediated by reduced chemokine/cytokine levels in the air pouch rather than by local cell death. Since numbers of T cells, B cells, memory T cells, and memory B cells were not enhanced by preinfection with either male or female schistosomes or worm pairs in our air pouch model, we cannot comment on the role of these cells in combating invading cercariae. Type IV hypersensitivity typically occurs at least 48 h after exposure to an antigen (26), thus it is quite possible that this reaction/recruitment time was not long enough to permit an adequate T or B cell reaction. Further steps are clearly required to address this issue.

Our data indicate that the immune suppression observed is mediated by the upregulation of anergy-related genes *Ctla4* and *Il10* in group mf. Both genes are known to play a role in granulomatous hyporesponsiveness (27). Since it is known that parasite eggs induce the expression of anergy-related genes, high expression levels of *Ctla4* and *Il10* in bisexually infected mice (group mf) are related to the presence of parasite eggs rather than to a synergistic effect of worm pairs. As recently shown, *Ctla4* expression levels were significantly elevated after preinfection with female schistosomes in a bisexually infection system using an extended preinfection period (14). Although not significant, there was an increase of *Il10* and *Ctla4* expression levels in spleens of group f mice compared to group m and uninfected mice in this study as well. These findings might indicate that anergy-related mechanisms are involved in a time-dependent manner. Moreover, we found an upregulation of *Il33* and its receptor, described as potent mediators of immune suppression in infectious or sterile inflammation (28, 29). The induction of anergy by *S. mansoni* worms has already been demonstrated (30). We present evidence here that the female worm is responsible for this phenomenon.

It has been reported that recurrent helminth infections cause hyporesponsiveness in dermal lymphocytes (10, 31) and the polarization of alternatively activated macrophages and dendritic cells, triggering a Th2 immune response (26, 27, 32, 33). The efficacy of Th2 responses in killing skin-penetrating cercariae has been shown in avian schistosomiasis. Moreover, the majority of *Trichobilharzia* spp cercariae die within a few days of exposure to murine skin. The dermal reactions of mice and humans to cercariae are described as allergic-type hypersensitivity and include immediate and late phase reactions, Th2-polarization, an increase in IgG1 and IgE levels, and histamine release (13, 34, 35). Higher production of Th2 cytokines in mice carrying male schistosomes in our infection model suggests that Th2 responses also contribute to the host’s defense.

Neutrophils and eosinophils efficiently kill parasites by producing elastase and hydrogen peroxide (9). We thus hypothesized that the strong recruitment of innate inflammatory cells observed in mice carrying male worms should result in a reduction of worm pairs and egg burden. In a two-step infection model, mice carrying male, female, or male and female schistosomes were bisexually reinfected after 6 weeks and analyzed 8 weeks later (14 weeks p.i.). It was indeed found that a strong recruitment of inflammatory cells correlates with a marked reduction in parasite burden in mice primarily infected with male cercariae. Inversely, preinfection with female cercariae did not lead to parasite reduction. As expected, the highest numbers of worm pairs and eggs were seen in group mf/MF, because of the double infection with mixed cercariae. Parasite burden in mice infected only once (–/MF) was higher than in mice preinfected with male schistosomes, despite once-infected mice displaying a strong dermal immune response. This makes sense since they had no opportunity to develop protective IgG1 and IgG2b antibody responses or to recruit macrophages into the skin prior to infection. Parasites not killed in the skin are often killed in the lungs. Experimental studies indicate that immunologically concealed adult worms might promote so-called antilarval concomitant immunity *via* the release of cross-reactive antigens (36).

The reduction of parasite loads in mice preinfected with male cercariae has a limited effect on hepatic fibrosis and granuloma size. The ratios of liver/body weight and spleen/body weight were also elevated to a comparable extent in –/MF, f/MF, and m/MF mice. Although tissue-entrapped eggs act as inflammatory stimuli, the principal driver of hepatic fibrosis progression are pro-fibrotic interleukins-4 and -13. At a certain level of infection (egg loads within the liver), Th2-mediated fibrosis becomes exaggerated and progresses independently from hepatic egg load or size of hepatic granulomas (37). IL-4 and IL-13 determine the inflammatory phenotype of egg-induced granulomas and IL-13 causes fibrosis (38). We conclude, therefore, that a reduction in parasite burden may not be sufficient for the treatment of hepatic damage, particularly hepatic fibrosis.

The results of the present study reveal how schistosomes differentially manipulate the host’s immune system when it comes to combating invading cercariae. Immunosuppression by helminths is a mechanism known to facilitate parasite survival and promote chronic disease progression. Though the host benefits from a reduction in tissue damage, immunosuppressive cytokines can also reduce vaccine responses (39) since the effect of a vaccine is not at least dependent on the host’s immune competence. We propose here that the female worm is a neglected player in the dampening down of the host’s immune defense mechanisms and is therefore a promising target for new immune modulatory strategies.

## Ethics Statement

Animal experiments were performed in strict accordance with the regulations of the German Society for Laboratory Animal Science and with the European health guidelines issued by the Federation of Laboratory Animal Science Associations. The protocol was approved by the local committee on animal care and use (7221.3-1.1-008/13, 7221.3-1-047/16, 7221.3-2-024/15-2). All efforts were made to minimize animal suffering.

## Author Contributions

MS, NK, and ER conceived and designed the experiments. MS, NK, FW, and SS performed the experiments. MS, NK, AR, CF, and ML analyzed the data. MS, NK, AR, ML, and ER contri-buted reagents/materials/analysis tools. MS, NK, AR, and ER wrote the paper.

## Conflict of Interest Statement

The authors declare that the research was conducted in the absence of any commercial or financial relationships that could be construed as a potential conflict of interest.
