# Supplementary material for: Host Defense Versus Immunosuppression: Unisexual Infection With Male or Female Schistosoma mansoni Differentially Impacts the Immune Response Against Invading Cercariae
Source: Front Immunol. 2018 Apr 24;9:861. doi: 10.3389/fimmu.2018.00861 (PMC5930291; doi:10.3389/fimmu.2018.00861)
Supplement: Supplementary file 1 [file Data_Sheet_1.docx]

**Supplementary information**

**Host defence versus immunosuppression: Unisexual infection with male or female *Schistosoma mansoni* differentially impact the immune response against invading cercariae**

Martina Sombetzki, Nicole Koslowski, Anne Rabes, Sonja Seneberg, Franziska Winkelmann, Carlos Fritzsche, Micha Loebermann, Emil C. Reisinger

**Supplementary Tables**

**Supplementary Table 1.** Antibodies and enzyme-linked immunosorbent assays used in this study:

| **Antibody** | **Reference number** | **clone** |
| --- | --- | --- |
| anti-CD45-PerCP | 103129 | 30-F11 |
| anti-CD11b-PE | 101207 | M1/70 |
| anti-CD11c-Alexa488 | 117313 | N418 |
| anti-Gr-1-PE-Cy7 | 108415 | RB6-8C5 |
| anti-F4/80-APC | 123115 | BM8 |
| anti-B220-PE-Cy7 | 103221 | RA3-6B2 |
| anti-CD3-APC | 100235 | 17A2 |
| anti-CD4-FITC | 100405 | GK1.5 |
| anti-CD8-APC-Cy7 | 100713 | 53-6.7 |
| anti-CD44-PE | 553134 | IM7 |
| anti-62L-APC-Cy7 | 557655 | 1D3 |
| anti-B220-APC-Cy7 | 552094 | RA3-6B2 |
| anti-PD-L2-APC | 564715 | MIH5 |
| anti-CD73-PE | 550741 | TY/23 |
| anti-CD80-PerCP-Cy5.5-APC | 559370 | L307.4 |
| IFN-γ, TNF-α, IL-1β, IL-12p70, IL-4, IL-5, IL-13, IL-10 | EPX110-20820-901 |  |
| RANTES, Eotaxin, MIP-1α, MIP-1β, MCP-1, CXCL-1. CXCL-2, CXCL-10 | EPX090-20821-901 |  |
| TGF-ß ELISA | 88-8350-22 |  |
| IL-10 | EPX01A-20614-901 |  |

**Supplementary figure legends**

**Supplementary figure 1. Experimental setup.** To evaluate the effect of a primary unisexual *S. mansoni* infection on injected Schistosoma mansoni (air pouch) cercariae or on a bisexual challenge infection, three independent experimental designs were performed: study design “air pouch”, study design “antibodies and gene expression” and study design “bisexual infection”.

**Supplementary figure 2: Female schistosomes suppress the recruitment of innate immune cells.**

Total cell counts (A), percentage of Gr1+ inflammatory monocytes (B), eosinophils (C), neutrophils (D) and macrophages/resident monocytes (E) were analyzed in air pouch exudates. Data from individual experiments are depicted as individual symbols, lines represent mean +/- SEM; n = 5 or 10 mice each group; *p<0.05, **p<0.01, ***p<0.001, n.s., not significant.

**Supplementary figure 3: Single sex or bisexual infection with *Schistosoma mansoni* has no impact on memory T- and memory B-cell recruitment following injection of cercariae into an air pouch.**

Percentage (A) or cell counts (B) of memory T-cells and percentage (C) or cell counts (D) of memory B-cells were analyzed in air pouch exudates. Data from individual experiments are depicted as individual symbols, lines represent mean +/- SEM; n = 5 or 10 mice each group; n.s., not significant.

**Supplementary figure 4: Serum biochemistry following bisexual challenge infection.** Serum levels of aspartate aminotransferase (AST), alanine aminotransferase (ALT) and alkaline phosphatase (AP) are presented as mean ± SD; *p<0.05, **p<0.01, ***p<0.001

**Supplementary Figures**

**Supplementary figure 1.**

**
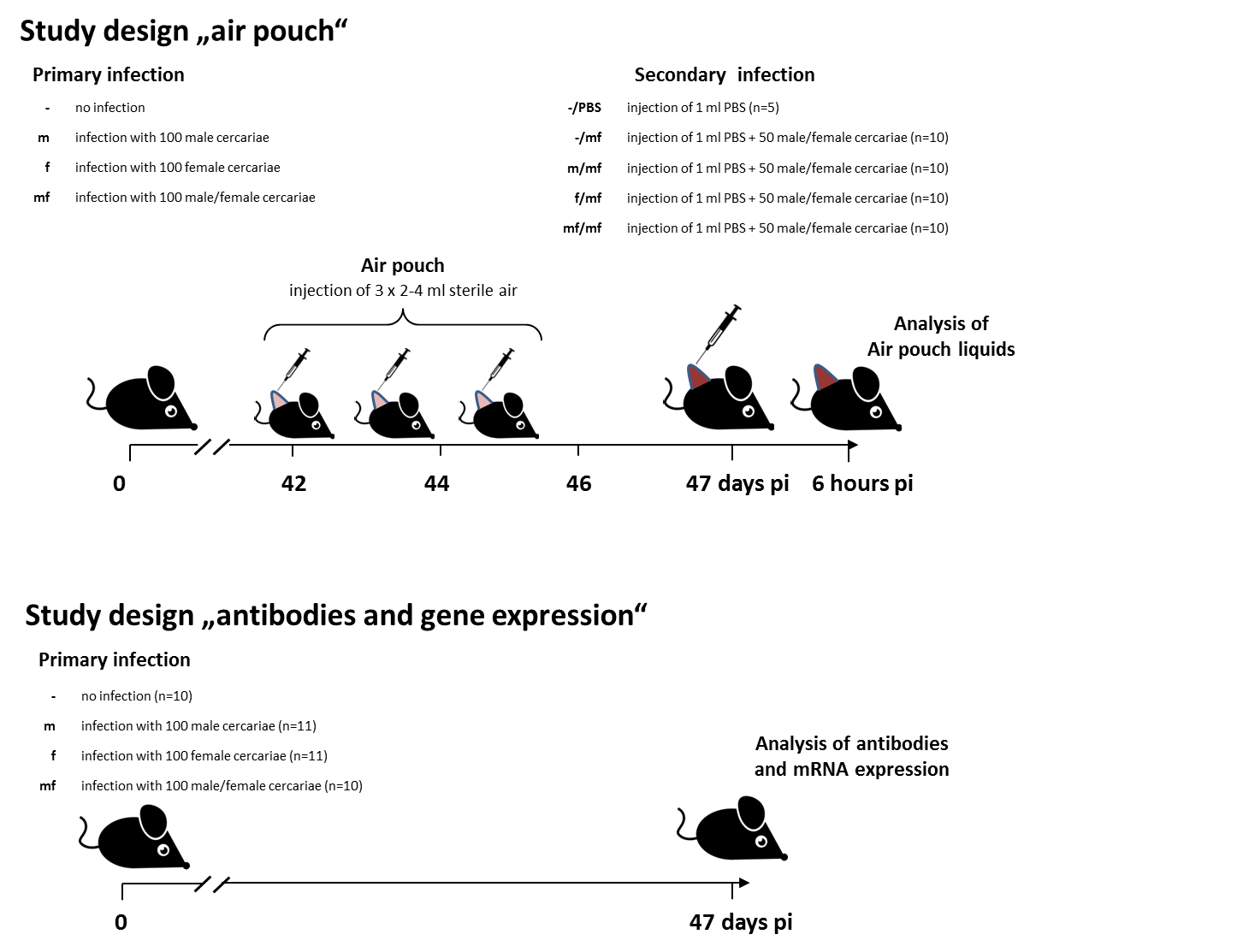
**

**
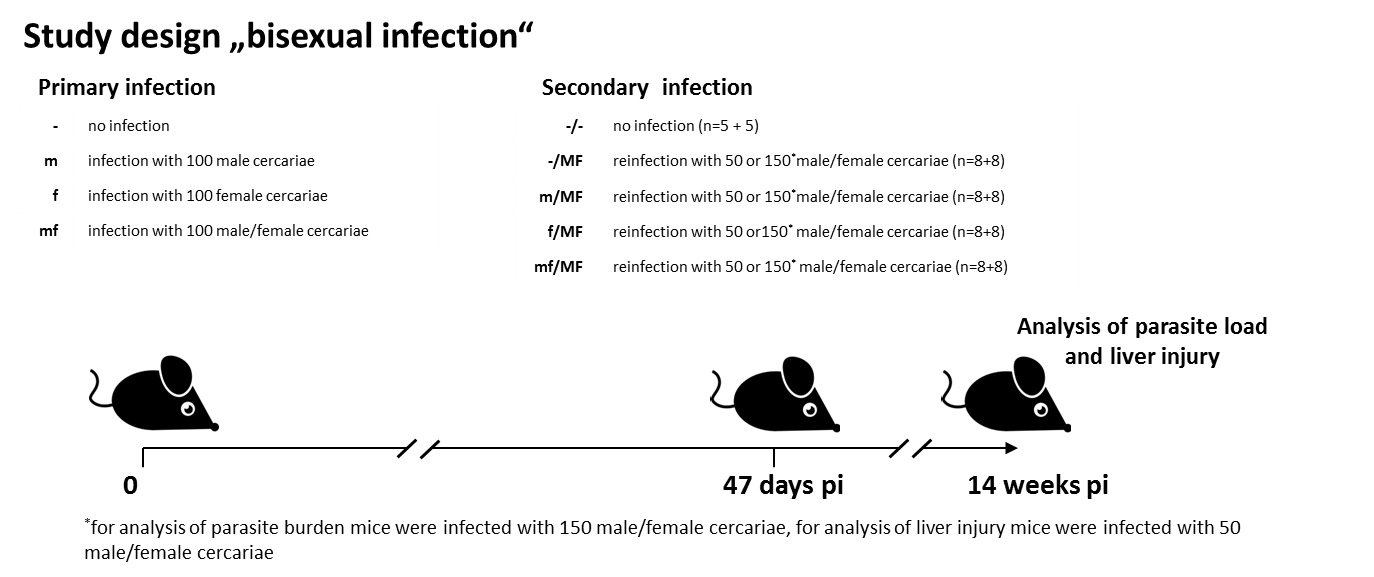
**

**Supplementary figure 2.**

**
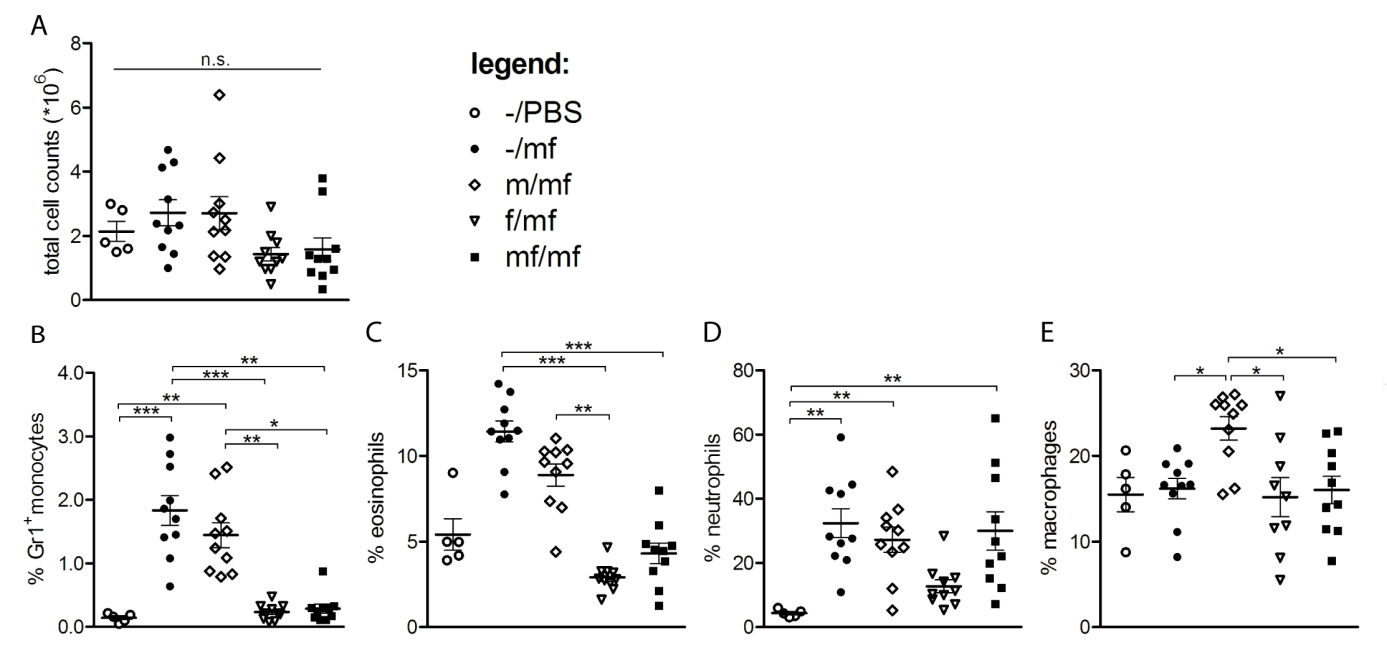
**

**Supplementary figure 3.**

**
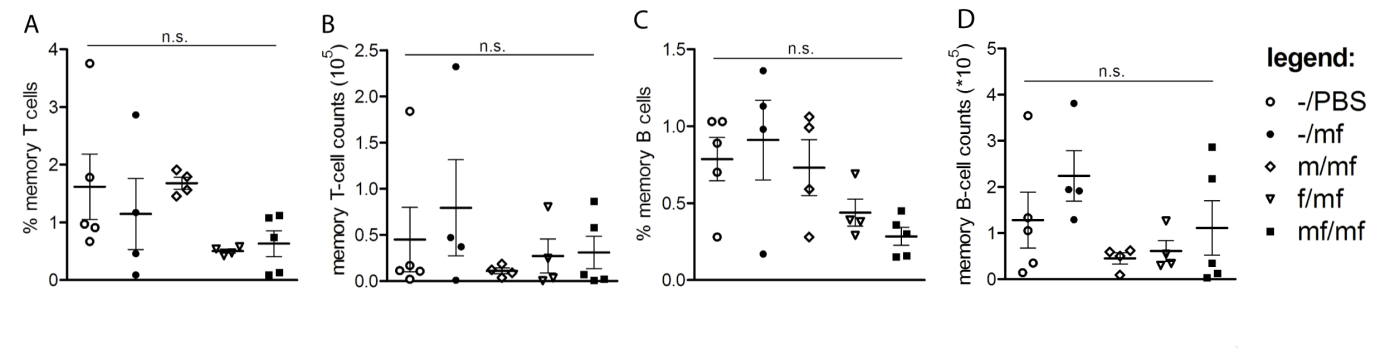
**

**Supplementary figure 4.**

**
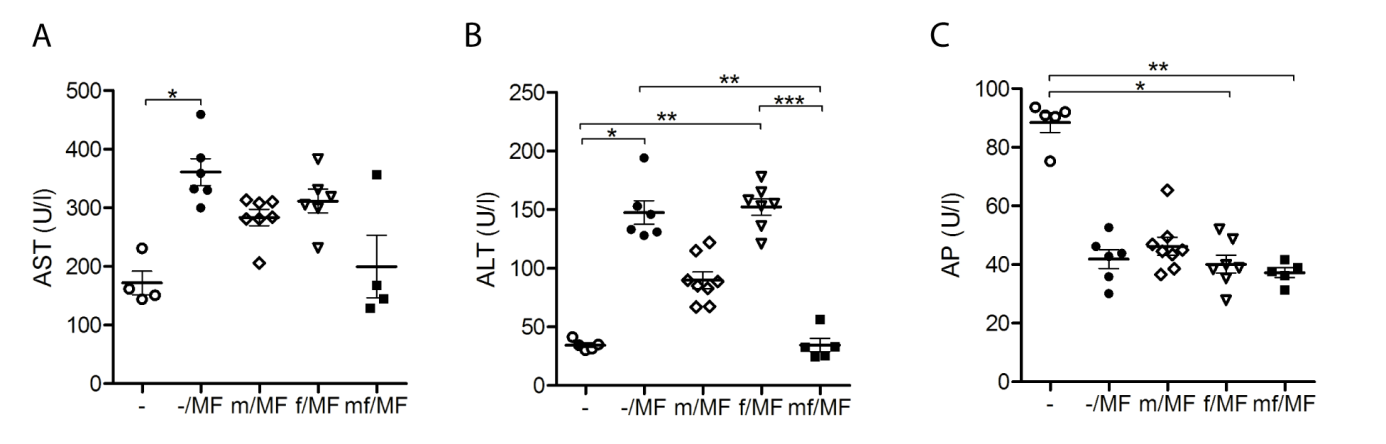
**
